# Host Transcriptional Profiles and Immunopathologic Response following *Mycobacterium avium* subsp. *paratuberculosis* Infection in Mice

**DOI:** 10.1371/journal.pone.0138770

**Published:** 2015-10-06

**Authors:** Min-Kyoung Shin, Hongtae Park, Seung Won Shin, Myunghwan Jung, Su-Hyung Lee, Dae-Yong Kim, Han Sang Yoo

**Affiliations:** 1 Department of Infectious Diseases, College of Veterinary Medicine, Seoul National University, Seoul, Korea; 2 Department of Microbiology, Gyeonsang National University School of Medicine, Jinju, Korea; 3 Department of Veterinary Pathology, College of Veterinary Medicine, Seoul National University, Seoul, Korea; 4 Institute of Green Bio Science and Technology, Seoul National University, Pyeongchang, Korea; Federal University of Pelotas, BRAZIL

## Abstract

Paratuberculosis or Johne’s disease is a chronic granulomatous enteropathy in ruminants caused by *Mycobacterium avium* subsp. *paratuberculosis* (MAP) infection. In the present study, we examined the host response to MAP infection in spleens of mice in order to investigate the host immunopathology accompanying host-pathogen interaction. Transcriptional profiles of the MAP-infected mice at 3 and 6 weeks p.i. showed severe histopathological changes, whereas those at 12 weeks p.i. displayed reduced lesion severity in the spleen and liver. MAP-infected mice at 3 and 6 weeks p.i. showed up-regulation of interferon-related genes, scavenger receptor, and complement components, suggesting an initial innate immune reaction, such as macrophage activation, bactericidal activity, and macrophage invasion of MAP. Concurrently, MAP-infected mice at 3 and 6 weeks p.i. were also suggested to express M2 macrophage phenotype with up-regulation of *Mrc1*, and *Marco* and down-regulation of *MHC class II*, *Ccr7*, and *Irf5*, and canonical pathways related to the T cell response including ICOS-ICOSL signaling in T helper cells, calcium-induced T lymphocyte apoptosis, and CD28 signaling in T helper cell. These results provide information which furthers the understanding of the immunopathologic response to MAP infection in mice, thereby providing insights valuable for research into the pathogenesis for MAP infection.

## Introduction

*Mycobacterium avium* subsp. *paratuberculosis* (MAP) is the causative agent of Johne’s disease, which is characterized by chronic granulomatous enteropathy, persistent diarrhea, progressive wasting, and potential death in ruminants [1,2]. Although domestic and free-ranging ruminants are the animals primarily vulnerable to MAP, MAP infection has also been reported in primates, rabbits, weasels, stoats, and foxes [3–5]. In addition, a possible link between MAP and Crohn’s disease, which is a type of chronic inflammatory bowel disease in humans, has been mentioned in several studies [6]. The presence of MAP has been reported in the intestine, blood, and breast milk of Crohn’s disease patients [7–11]. Many MAP studies examining host-pathogen interactions have used cattle, which are the natural hosts for MAP. However, use of ruminants in experimental challenge models for Johne’s disease is complicated by the long experimental period required, wherein the animals have to be maintained until they develop signs of the disease [12–15]. Although a bovine model that can be used to study all stages of MAP infection has not been reported to date, a baby goat model has been recently used for studying vaccine strain of MAP [16].

A murine MAP challenge model has been developed for the early screening of vaccine candidates and preliminary analysis of pathogenesis [15]. As mice are not a natural host for MAP, a murine model has its limitations for reproducing typical features of Johne's disease, such as diarrhea and severe intestinal lesions, understanding granuloma development and progression in response to mycobacterial infection, and establishing mycobacterial latency [15,17,18]. Nevertheless, the availability of immunological reagents and the variable genetic background of mice have rendered murine models suitable for studying the pathogenic mechanisms of MAP [15,19–21]. In addition, granulomatous lesion formation and cytokine (e.g., IL–1, IL–6, TNF, and INF-γ) secretion, which represent the histopathological and immunological characteristics of MAP infection, respectively, have also been observed in mice [15,19,20,22]. In a previous study, intraperitoneal (IP) injection of MAP was found to reproduce infection in all inoculated mice (100%), whereas oral inoculation induced MAP infection in only 58% of mice [15,23]. Therefore, although oral route is the natural route of MAP infection in cattle, IP injection has been frequently utilized in MAP infection of mice. The number of lesions and degree of bacterial colonization differed amongst mouse strains, with BALB/c and C57BL/6 strains being susceptible to infection, and C3H strain being more resistant [15,19,23]. As previous reports on murine experimental models have been focused on histopathological and immunological features, additional works, such as analyses of gene expression profiles using microarrays, are needed [15,19,20,22]. Traditionally, host immune responses following mycobacterial infection have been studied in macrophage, as mycobacteria persist within macrophages using a variety of immune evasion strategies such as preventing recognition of infected macrophages by T cells, and evading macrophage-mediated killing through blockage of phagosome acidification and maturation as well as evasion of nitric oxide and related reactive nitrogen intermediates [24–27]. Actually, many studies have used approached to described the gene expression profiles of macrophages and splenocytes during *Mycobacterium tuberculosis* infection [28–30], as well as bovine macrophages [1,31,32] during MAP infection, but there has been no report on MAP infection in a murine model, other than transcriptional profiles in murine macrophage, RAW 264.7 cells, as our previous study [33].

In the present study, we analyzed pathological changes and transcriptional profiles induced in the murine spleens, wherein much of the cross-talk between innate and adaptive immune cells occurs, following MAP infection [34,35]. To the best of our knowledge, this is the first report describing the transcriptional profiles and immunopathology associated with the host anti-mycobacterial response during MAP infection in the spleens of mice.

## Results

### Pathological findings in mice after MAP challenge

**Fig 1:**
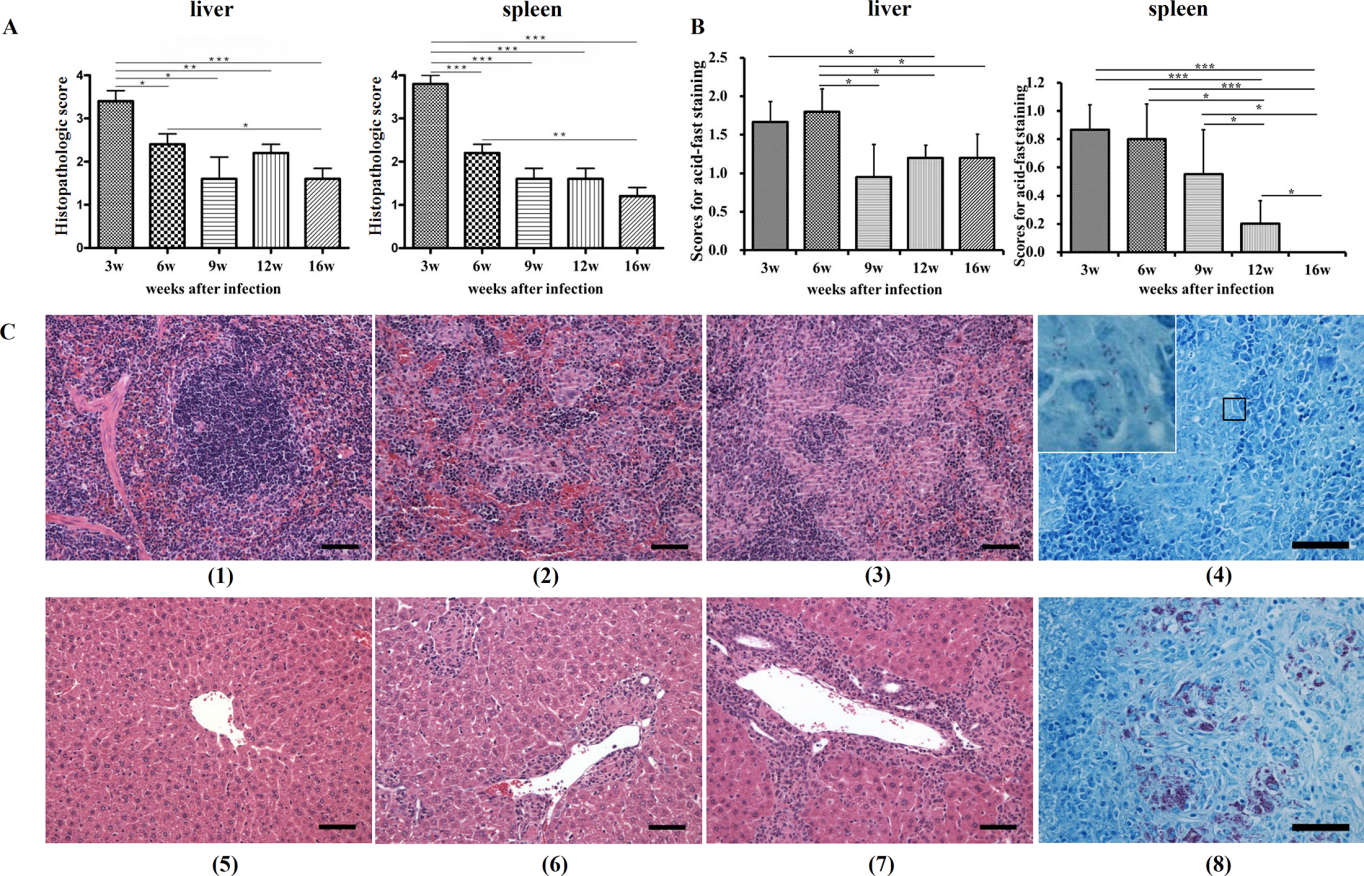
Pathological observations. **A)** histopathology scores, **B)** scores for acid-fast-positive bacilli in the liver and spleen, and **C)** representative histopathology and acid-fast stain of the spleen and liver, HE, bar = 100μm (original magnification x400; inset, x1000) Note spleen with (1); normal, (2); +2 and, (3); +4 degree of inflammation. Also note liver with (5); normal, (6); +2, and, (7); +4 degree of inflammation. Also note myriads of acid-fast positive bacilli in the liver (8) with scant numbers of bacilli in the spleen (4). Values are represented as mean ± SE, and were analyzed by Student's *t*-test for mean weight and histopathology and the Mann Whitney *U*-test for acid-fast bacilli. (***, *p* < 0.001; **, *p* < 0.01; *, *p* < 0.05).

Histopathological changes were observed during the experimental period in spleens and livers of the MAP-infected mice. In the spleen, the normal splenic architecture was completely effaced due to severe infiltration of a large number of macrophages and lesser number of lymphocytes with obliteration of the normal white and red pulp structure at 3 weeks p.i. (Fig 1C(3)). Moderate effacement of the normal splenic architecture due to infiltration of a moderate number of macrophages with compression to destruction of the splenic white pulp at 6 weeks p.i. and mild infiltration of macrophage in the parafollicular areas.at 12 weeks p.i. were noted in the infected spleen ((Fig 1C(2) and 1C(1)). The mean lesion scores at 6, 9, 12, and 16 weeks p.i. were significantly lower than those at 3 weeks p.i. (p < 0.001). In addition, the lesion score at 6 weeks p.i. was higher than that at 16 weeks p.i. (*p* < 0.01) (Fig 1A). In the liver, portal and periportal hepatitis along with lymphocyte, plasma cell, and macrophage infiltration were observed (Fig 1C(5), 1C(6) and 1C(7)) and the lesion severity decreased with time. Large numbers of macrophages and lymphocytes infiltrated around portal triads and in the hepatic parenchyma at 3 weeks p.i.. Moderate numbers of lymphocytes, and small numbers of macrophages, lymphocytes, and plasma cells infiltrated around portal triads at 6 and 12 weeks p.i., respectively. The mean lesion score at 3 weeks p.i. was significantly higher than that at 6- (*p* < 0.05), 9- (*p* < 0.05), 12- (*p* < 0.01), and 16-weeks p.i. (*p* < 0.001) time points, and the score at 6 weeks p.i. was also higher than that at 16 weeks p.i. (*p* < 0.05), similar to the pattern observed in the spleen (Fig 1A). Control mice had no lesions in either the spleen or liver over the infection time course (data not shown).

Acid-fast staining revealed a myriad of acid-fast positive bacilli mainly in the cytoplasm of macrophages (Fig 1C(4)). Compared to the liver, bacterial load was much lower in the spleen regardless at all time points after infection (Fig 1C(8)). Although the mean score for acid-fast bacilli gradually decreased and acid-fast-positive bacilli were not detectable in the spleen 16 weeks p.i., MAP was detected in the liver throughout the experimental period (Fig 1B).

### Determination of differentially expressed genes

**Fig 2:**
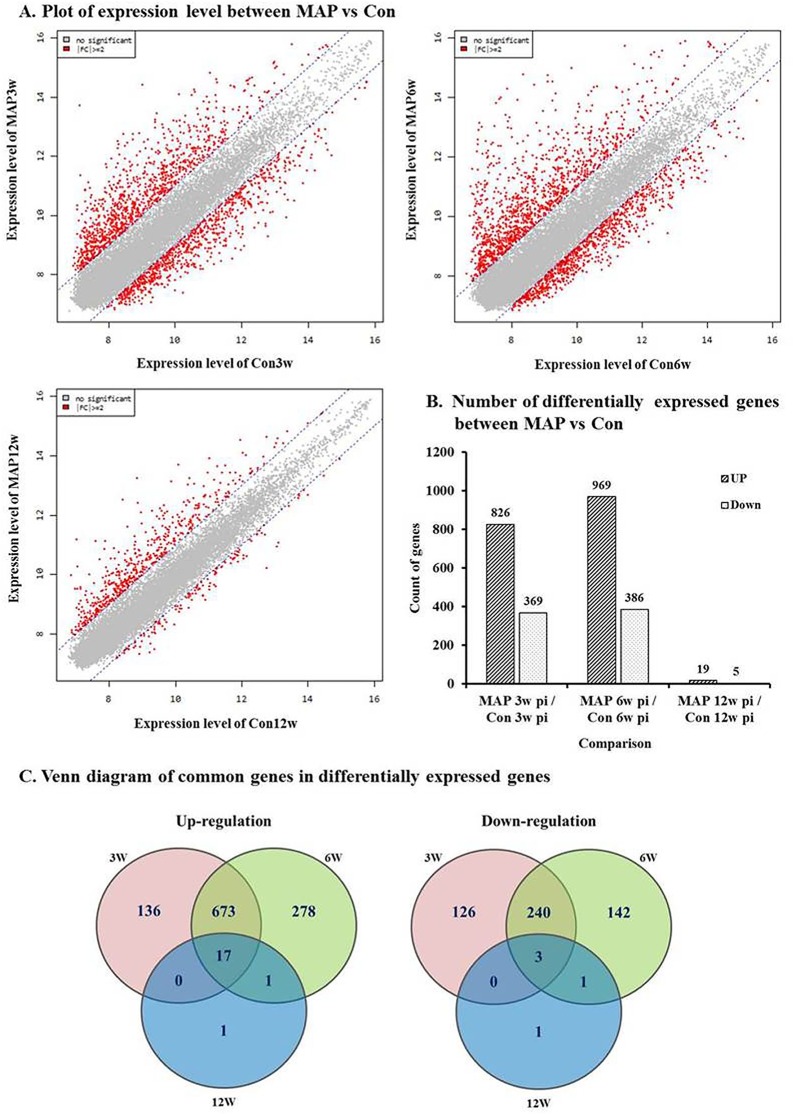
Comparison of gene expression levels between control and MAP-challenged mice. **A)** Scatter plots comparing gene expression levels between MAP-challenged and control groups at 3, 6, and 12 weeks post-infection. Red dots indicate an expression level change of |fold change| ≥ 2. The expression levels were calculated by using the base–2 logarithm of the normalized hybridization signals from each sample. **B)** Numbers of genes with altered expression levels during this experiment. **C)** Venn diagram showing overlapping genes that were significantly up- or down-regulated in MAP-challenged mice at 3, 6, and 12 weeks post infection (*p* < 0.05, |fold change| ≥ 2).

The present study used microarrays (Illumina Mouse WG–6 v2 Expression BeadChip) for analysis of gene expression in MAP-infected and control mice spleens. We analyzed altered transcription in spleens of MAP-infected mice at 3, 6, and 12 weeks p.i., which were time points showing severe histopathological changes (3 and 6 weeks p.i.) or reduced lesion severity (12 weeks p.i.), using scatterplots showing median values for normalized hybridization signals (Fig 2A). Among the 31,059 genes analyzed, 1195 (3.85%), 1355 (4.36%), and 24 (0.08%) genes were differentially expressed in MAP-infected mice compared to control mice at 3, 6, and 12 weeks p.i., respectively. Among these, 826, 969, and 19 genes were up-regulated and 369, 386, and 5 genes were down-regulated in MAP-infected mice at 3, 6, and 12 weeks p.i., respectively (Fig 2B). Fig 2C shows the number of genes commonly up- or down-regulated among the differentially expressed genes in MAP-infected mice at 3, 6, and 12 weeks p.i. In total, 17 and 3 shared genes were up- or down-regulated, respectively, during the experimental period. In particular, many genes showed similarly altered expression in MAP-infected mice at 3 and 6 weeks p.i., with 673 and 240 genes being up- or down-regulated, respectively (Fig 2C). Hierarchical clustering of differentially expressed genes also showed a correlation between MAP-infected mice at 3 and 6 weeks p.i. (data not shown). The raw files and normalized datasets are available at Gene Expression Omnibus (GEO) (http://www.ncbi.nlm.nih.gov/geo website) under accession number GSE62836.

The top 10 up- and down-regulated genes are listed in S1 Table. Up-regulated genes induced by MAP infection were found to be related to immunity and defense (*Saa3*, *Gbp1*, *S100a8*, *S100a9*, *Lcn2*, *Ctse*, and *Prdx2*), signal transduction (*Kel*, *S100a9*, and *Gdf3*), lipid metabolism (*Abcg4*), other metabolic processes (*Kel*, *Trim10*, *Car1*, *Tal1*, *Gclm*, *Ear4*, and *Ctse*), cell structure and motility (*Ank1*, *Gypa*, and *S100a8*), and transport (*Ltf*, *Lcn2*, *Hbb-b1*, and *Rhag*). Most of the highly up-regulated genes were related to immunology and metabolism. The infected mice at 3 and 6 weeks p.i. were suspected as being in the early stage of MAP infection based on features such as a high expression of acute phase protein (*Saa3*), IFN-γ inducible protein (*Gbp1*), and *S100a8* and *S100a9* (also termed myeloid-related proteins (MRP)8 and MRP14).

The most strongly down-regulated genes played roles in immunity and defense (*Cfd*, *Gm459*, *Vpreb3*, *LOC384415*, *Fcer2a*, and *Ccl21c*), other metabolic processes (*Cfd*, *Gm459*, *Vpreb3*, *LOC384415*, *Cd79b*, *Chst3*, and *Psmb1*), signal transduction (*Cd79b*, *Fcer2a*, *Ccl21c*, *Igfbp5*, *Cd37*, and *Ebi2*), and transport, (*Cd79b* and *Chst3*). Most highly down-regulated genes were associated with signal transduction, immunity and defense, and metabolism.

### Biological functions of differentially expressed genes in MAP-infected mice

**Fig 3:**
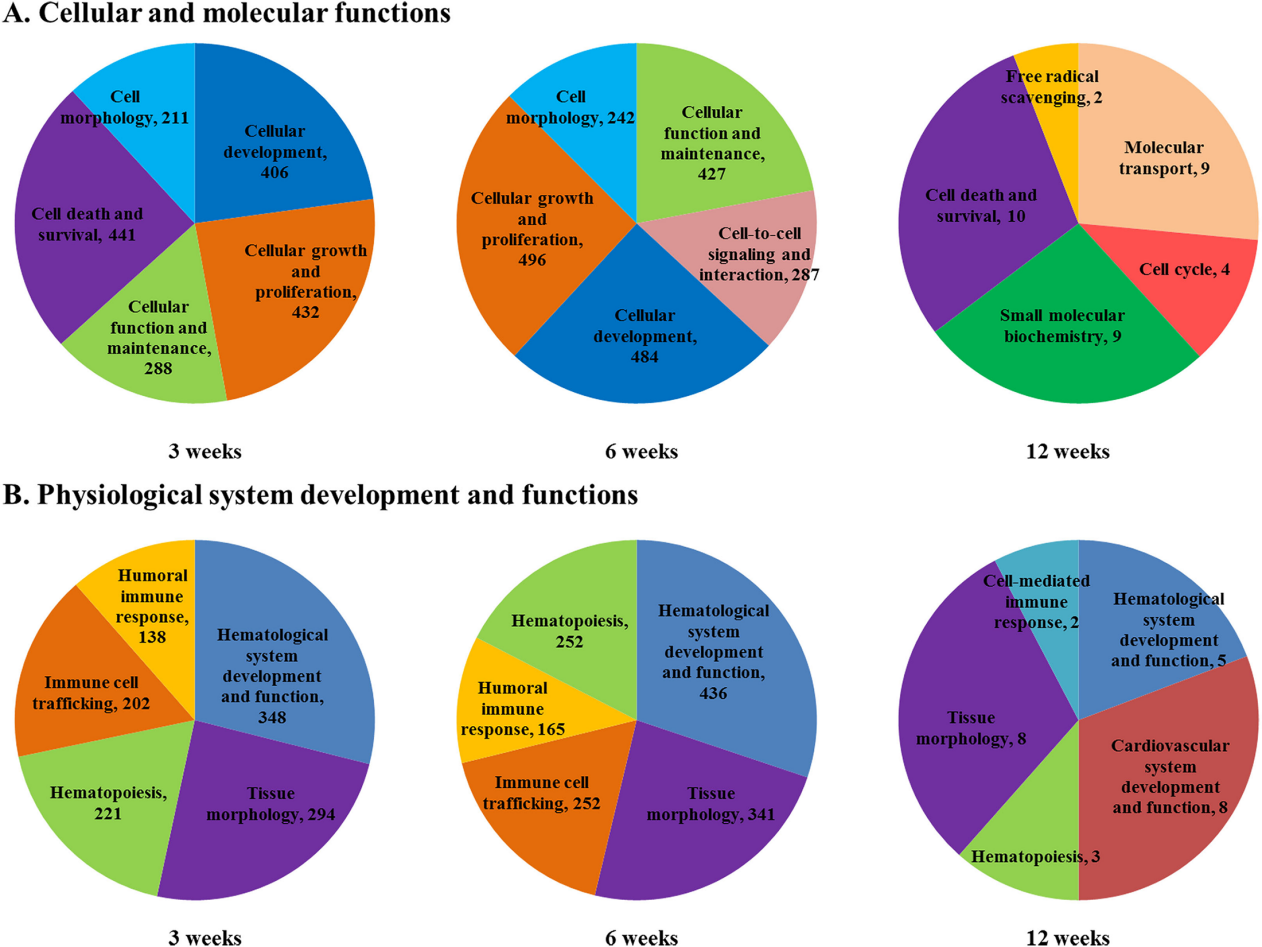
Functional characterization of differentially expressed genes in MAP-challenged mice. **A**) Cellular and molecular functions and **B)** physiological system development and functions of differentially expressed genes in MAP challenged mice at 3, 6, and 12 weeks post-infection. Top five functions were identified for each analysis by IPA.

In total, 1215, 1414, and 27 among 1729, 1968, and 29 up- and down-regulated transcripts at 3, 6, and 12 weeks p.i., respectively, were mapped to molecules in the Ingenuity Knowledge Base. As described above, the differentially expressed genes at 3 and 6 weeks p.i. were in the same categories and presented similar numbers as those associated with cellular and molecular functions and physiological system development and functions (Fig 3). Genes were categorized by cellular and molecular functions including cell death and survival, cellular growth and proliferation, cellular development, cellular function and maintenance, and cell morphology, especially showing cell death and survival at 3 weeks p.i., but cell-to cell signaling and interaction at 6 weeks p.i. (Fig 3A). In addition, the genes were categorized as playing roles in physiological system development and functions including hematological system development and function, tissue morphology, hematopoiesis, immune cell trafficking, and humoral immune responses (Fig 3B). At 12 weeks p.i., the related cellular and molecular functions and physiological system development and functions included a small number of genes and different categories. In physiological system development and functions, humoral immune response was categorized in the differentially expressed genes at 3, and 6 weeks p.i., whereas cellular mediated immune response was associated with those at 12 weeks.

### Canonical pathway analysis

The top canonical pathways for the differentially expressed genes at 3, 6, and 12 weeks p.i. are reported in Table 1. Most of the pathways at 3 and 6 weeks p.i. were associated with the immune system. As shown in Fig 2, most of the genes showing altered expression at 12 weeks p.i. overlapped those at 3 and 6 weeks p.i. In addition, the top canonical pathways included a very low number of differentially expressed genes at 12 weeks p.i. (data not shown). Therefore, we only focused on the canonical pathways at 3 and 6 weeks p.i. Although most genes related to heme biosynthesis and estrogen-mediated S-phase entry were up-regulated at 3 weeks p.i., those within other pathways were down-regulated. In particular, the genes whose products played roles in the top five canonical pathways were down-regulated at 3 weeks p.i. Some of these genes playing roles in multiple pathways related to the T cell immune response (ICOS-ICOSL signaling in T helper cells, calcium-induced T lymphocyte apoptosis, and CD28 signaling in T helper cells), were down-regulated at 3 and 6 weeks p.i. In addition to *Cd28* and *ICOSL*, their downstream genes such as *LCK*, *CSK*, *ZAP70*, *IP3R*, *IkB*, *NFkB* and *NFAT* were down-regulated following MAP infection, suggesting that ICOS-ICOSL signaling in T helper cells and CD28 signaling in T helper cells is reduced following infection.

**Table 1:** Top five canonical pathways involving the genes that were differentially expressed after MAP infection.

	Canonical pathway	p-value	Genes/Total*	Up/Down
**3 weeks p.i.**	Calcium-induced T lymphocyte apoptosis	6.35E-09	18/71	1/17
	Heme Biosynthesis II	2.04E-07	7/24	7/0
	Estrogen-mediated S-phase entry	3.41E-07	10/28	10/0
	icos-icosL signaling in T helper cells	5.98E-07	21/126	1/20
	CD28 signaling in T helper cells	2.37E-06	21/136	1/20
**6 weeksp.i.**	icos-icosL signaling in T helper cells	6.69E-09	26/126	1/25
	Alerted T cell and B cell signaling in Rheumatoid arthritis	3.53E-08	22/100	4/18
	Calcium-induced T lymphocyte apoptosis	7.5E-08	18/71	1/17
	CD28 signaling in T helper cells	6.38E-07	24/136	1/23
	Role of NFAT in regulation of the immune response	1.15E-06	30/200	8/22

* Genes/Total = number of differentially expressed genes (2-fold change; p < 0.05) out of total genes associated with the canonical pathway according to IPA analysis.

### Gene networks of differentially expressed genes in MAP-challenged mice

**Fig 4:**
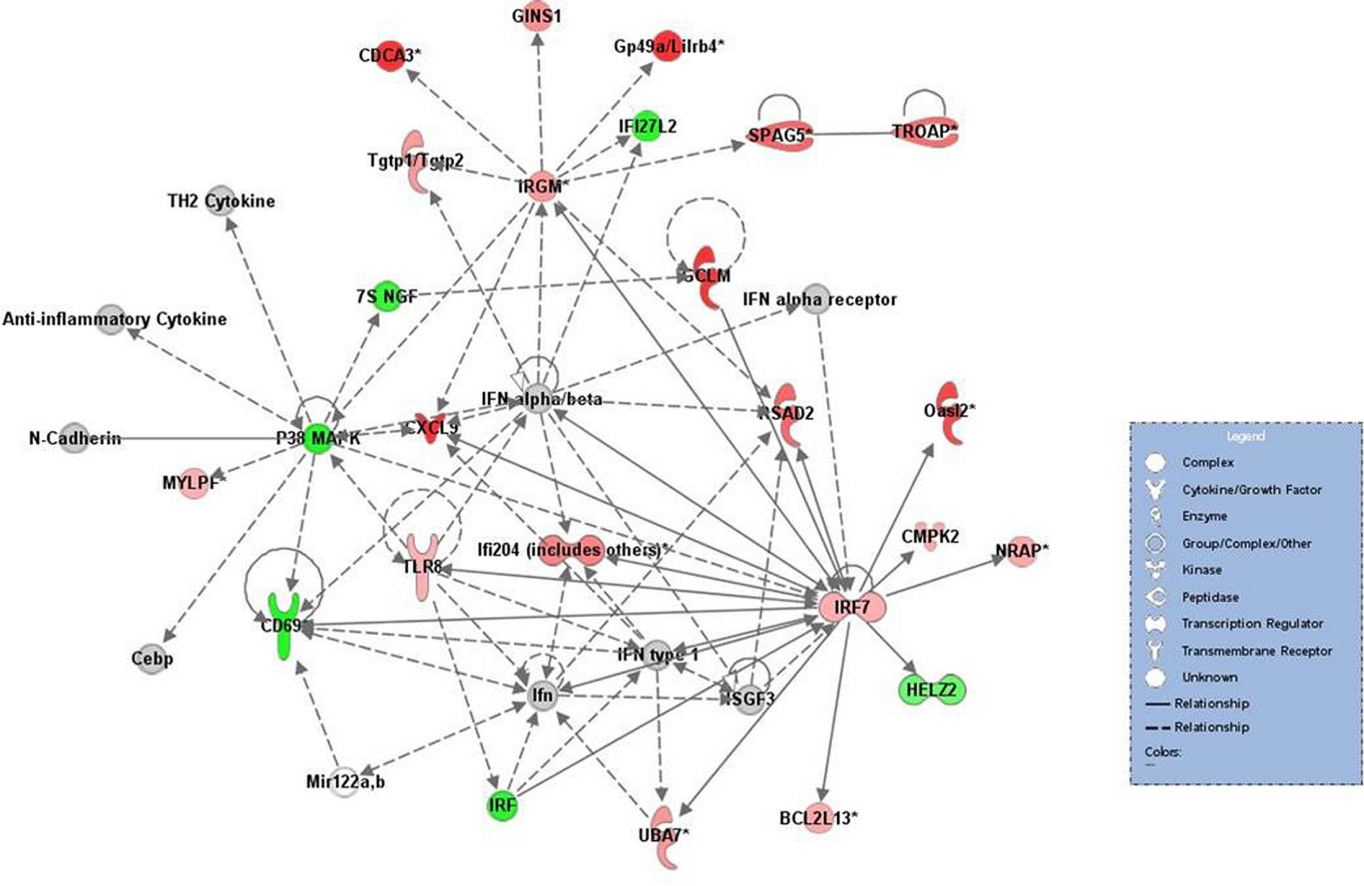
Representative network of the genes with altered expression in MAP-challenged mice at 3 weeks p.i. Network of down-regulated genes at 6 weeks p.i. Individual nodes represent proteins with relationships represented by edges. Nodes were colored to indicate changes in gene expression, with red indicating up-regulation, green indicating down-regulation, and white indicating that the gene/factor was not differentially expressed. Solid lines indicate a direct interaction, and dotted lines indicate an indirect interaction. Arrows indicate directional relationships.

Twenty-five networks were identified within the Ingenuity Knowledge Base among the differentially expressed genes at 3 or 6 weeks pi. As shown in Fig 4, the representative network for the identified genes at 3 weeks p.i. showed enrichment of factors associated with endocrine system disorders, gastrointestinal disease, and immunological diseases. The gene products associated with immunological diseases included the immunity-related GTPase family M protein (*Irgm1*, 2.2-fold up-regulation); interferon regulatory factor (*Irf3*, 1.6-fold; *Irf4*, 3.3-fold; *Irf5*, 1.7-fold down-regulation; *Irf7*, 2.1-fold up-regulation); Toll-like receptor 8 (*Tlr8*, 2.1-fold up-regulation); Cluster of differentiation 69 (*Cd69*, 3.4-fold down-regulation); and chemokine (C-X-C motif) ligand 9 (*Cxcl9*, 8.4-fold up-regulation). In particular, *Irf7*, in contrast to other *Irfs*, was up-regulated, indicating a relationship with *Tlr8*, *Cd69*, *Cxcl8*, *Irgm*, and *IFN*. Similar to *Irf7*, *Cxcl*9, *Irgm*, and *Tlr8* were up-regulated, thus resulting in an innate immune response against intracellular pathogens.

**Fig 5:**
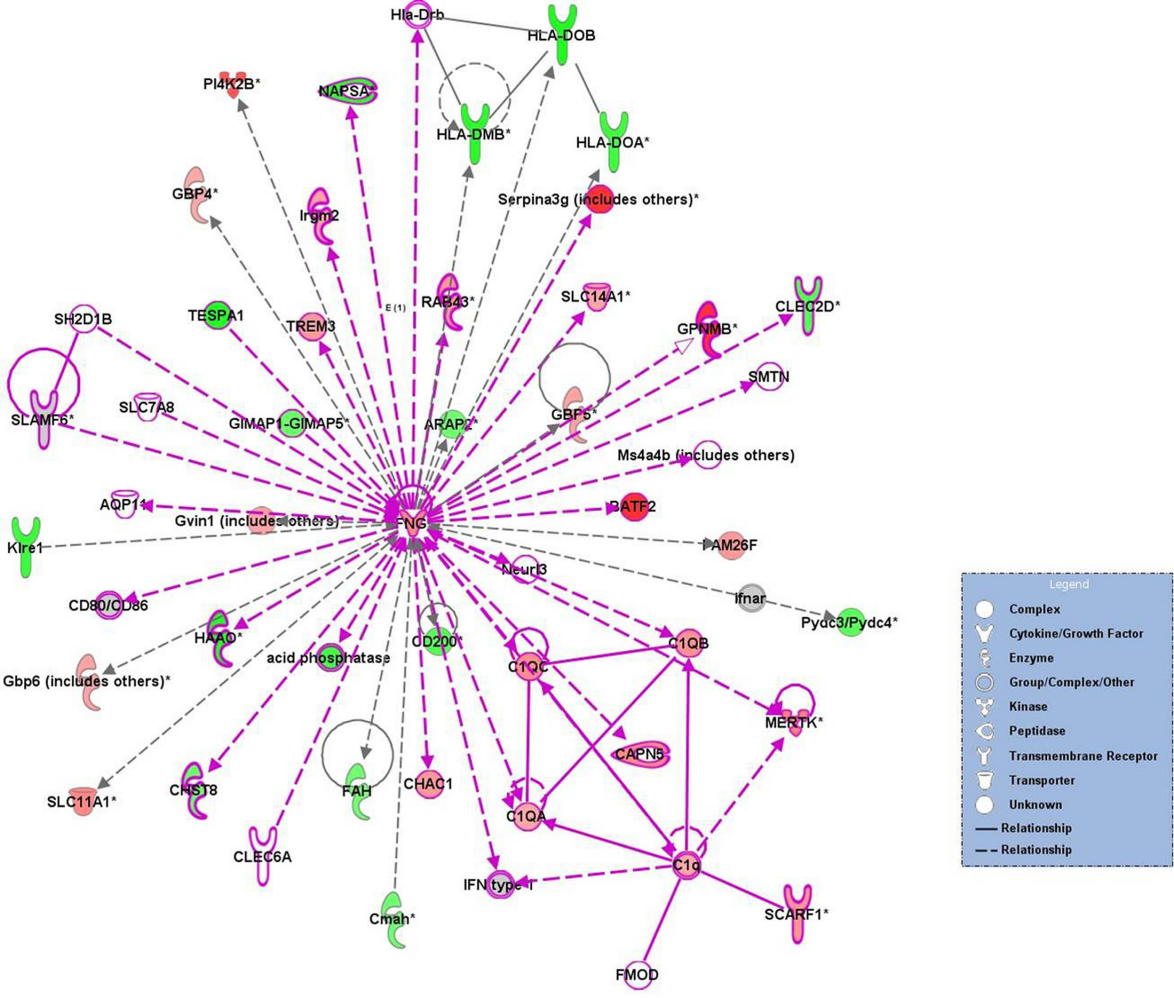
Representative network of the genes with altered expression in MAP-challenged mice at 3 and 6 weeks p.i. Two networks showing common genes and functions at 3 and 6 weeks p.i. were merged. The purple line indicates overlapped genes and relationships between 3 and 6 weeks p.i. Individual nodes represent proteins, with relationships represented by edges. Nodes are colored to indicate changes in gene expression, with red indicating up-regulation, green indicating down-regulation, and white indicating that the gene/factor was not differentially expressed. Solid lines indicate a direct interaction and dotted lines indicate an indirect interaction. Arrows indicate directional relationships.

Two networks that showed common genes and functions between 3 and 6 weeks p.i., were merged to investigate the relationships among the differentially expressed genes. The representative network of the differentially expressed genes at 3 and 6 weeks p.i. showed enrichment for factors associated with endocrine system disorders, gastrointestinal disease, immunological diseases, developmental disorders, and hereditary disorders (Fig 5). This network contained *IFN-γ* (2.4- and 1.9-fold up-regulation at 3 and 6 weeks p.i., respectively) and genes encoding complement components including *C1q* (*C1qa*, 2.3- and 3.9-fold up-regulation; *C1qb*, 2.9- and 5.1-fold up-regulation; *C1qc*, 3.0- and 4.9-fold up-regulation at 3 and 6 weeks p.i., respectively); transmembrane receptors such as scavenger receptor class F, member 1 (*Scarf1*, 2.8- and 3.1-fold up-regulation at 3 and 6 weeks p.i.); triggering receptor expressed on myeloid cells 3 (*Trem3*, 2.7- and 4.7-fold up-regulation at 3 and 6 weeks p.i., respectively); major histocompatibility complex class II molecules (*Hla-dmb*, 3.2- and 2.3-fold down-regulation; *Hla-doa*, 2.9- and 2.3-fold down-regulation; *Hla-dob*, 5.5- and 4.0-fold down-regulation at 3 and 6 weeks p.i., respectively); C-type lectin domain family 2, member D (*Clec2d*, 2.6- and 2.1-fold down-regulation at 3 and 6 weeks p.i., respectively); killer cell lectin-like receptor subfamily E, member 1 (*Klre1*, 2.9-fold down-regulation at 3 weeks p.i.); and guanylate binding proteins (*Gbp5*, 2.4- and 1.7-fold up-regulation; *Gbp6*, 3.7- and 2.6-fold up-regulation at 3 and 6 weeks p.i.).

### Validation of microarray data

**Fig 6:**
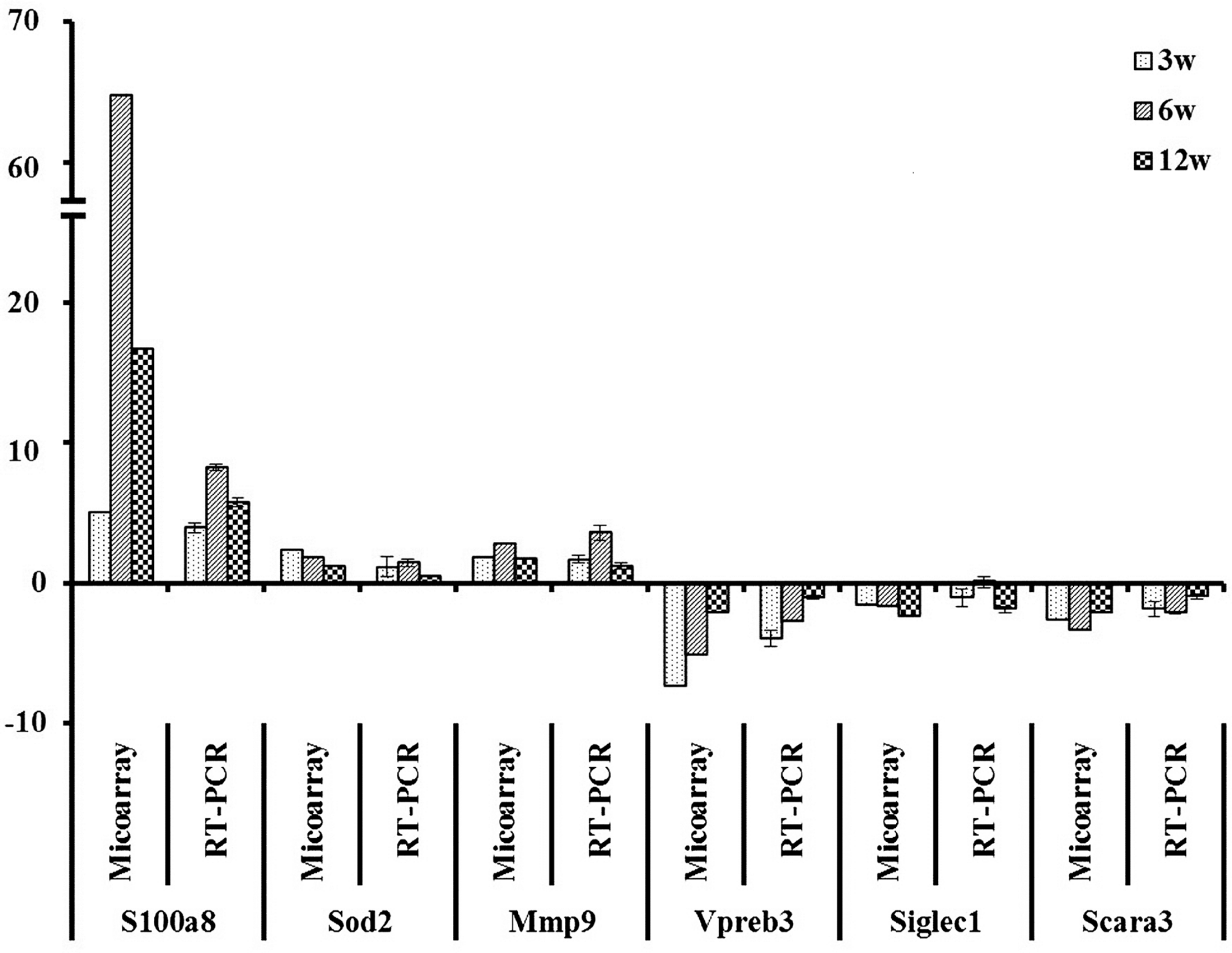
Validation of microarray data by quantitative RT-PCR. Relative expression level was normalized to the *GAPDH* expression level relative to control group by the 2^-ΔΔCT^ method.

Microarray results for six genes with altered expression were validated using qPCR. The *S100a8*, *Sod2*, and *Mmp9* genes from the up-regulated gene group as well as *Vpreb3*, *Siglec1*, and *Scara3* genes from the down-regulated gene group were randomly selected for qPCR (Table 2). The same RNA samples used for the microarray were used for qPCR. The Log2 fold change data for qPCR and microarray were analyzed for validation. The correlation coefficient between the two analyses was 0.807 (p < 0.001). Although the microarray data for some genes such as *S100a8* and *Vpreb3* showed more fluctuation (increase or decrease) compared to the qPCR data, the differential expression of all selected genes was validated, as the qPCR results showed the same trend, with respect to up-regulation or down-regulation (Fig 6).

**Table 2:** Primers used for qRT-PCR.

Accession No.	GeneSymbol		Sequence (5'->3')	Size (bps)
NM_013650.2	*S100a8*	Forward	CGAGGAGTTCCTTGCGATGG	84
		Reverse	CTAGGCCAGAAGCTCTGCTAC	
NM_013671.2	*Sod2*	Forward	GTGTCTGTGGGAGTCCAAGG	81
		Reverse	GCAGGCAGCAATCTGTAAGC	
NM_013599.2	*Mmp9*	Forward	GTCCAGACCAAGGGTACAGC	107
		Reverse	ATACAGCGGGTACATGAGCG	
NM_009514.4	*Vpreb3*	Forward	CTAGGTCGGCCTTTCTGCTT	121
		Reverse	AAGACTGAGAACGCGTCAGG	
NM_011426.2	*Siglec1*	Forward	CCGCATTGCAGCCATAAGTC	77
		Reverse	TGGCAATAGCTGTGTCTGGG	
NM_172604.3	*Scara3*	Forward	AAACAGCTCACCTCCCCCTA	117
		Reverse	GGTAGCTATCCCTTCCCCCA	

## Discussion

Despite the induction of strong host immune responses, mycobacteria can evade host immunity and cause persistent infection. The bacteria can survive and proliferate in host macrophage inside granulomas, thus leading to the development of immunopathology in gut or other tissues and shedding from the feces in the case of MAP infection [1,36,37]. The survival of the bacteria has been believed to be succeeded by various mechanisms including inhibition of phagosome maturation and suppression of immune-regulatory pathways [1,38,39]. Additionally, in the early to middle stages of mycobacterial infection, Th1 type immune responses are dominant and play key roles in the host anti-mycobacterial responses [40,41]. Switch from the Th1 to Th2 type immune response occurs concomitantly with a progression of MAP infection in clinical disease development, which is referred to as the classical switch profile [42,43].Up to now, analysis of the immunopathology has not been performed in mice using both transcriptional profiles and histopathological observations. Therefore, the current study may provide an understanding of immunopathological changes presenting MAP-host interactions.

In the present study, we investigated pathological changes and changes in the transcriptional profile in mice infected with MAP. We specifically analyzed altered transcription in spleens of MAP-infected mice at 3 and 6 weeks p.i. showing severe histopathological changes, and 12 weeks p.i. indicating reduced lesion severity. The number of differentially expressed transcripts and their relative fold change at 3 and 6 weeks p.i. were markedly higher than those at 12 weeks p.i. In addition, similar expression patterns at 3 and 6 weeks p.i. were observed for certain genes and the dendrogram as indicated by the venn diagram or hierarchical clustering, respectively. Biological functions and canonical pathways were also similarly categorized in MAP-infected mice at 3 and 6 weeks p.i. by the Ingenuity knowledge base. Among the top up- or down- regulated genes, some highly up-regulated genes were related to immunology and metabolism. Serum amyloid proteins (*Saa3*), which are induced during tissue injury, infection and inflammation, has been detected during murine tuberculosis, and in human tuberculosis patients [44]. *Gbp1* is related to cell-autonomous immunity against listerial or mycobacterial infection in macrophages as an IFN-γ inducible protein [45]. MRP8 and MRP14 produced by activated phagocytes are released during mycobacterial infection *in vitro* and *in vivo* [46,47]. In various reports, the expression of MRP8 and MRP14 by infiltrating cells showed a strong relationship with the inflammatory process [46,48–50]. In addition, most highly down-regulated genes were found to be associated with signal transduction, immunity and defense, and metabolism. In particular, down-regulated genes such as *LOC384415*, *Gm459*, *Vpreb3*, *Blk*, and *Cd79b*, were related to B-cell- and antibody-mediated immunity.

Integrins are involved in adhesion and penetration of intracellular pathogens and cell adhesion to components of the extracellular matrix such as laminin, collagen I, and fibronectin [51]. No significant difference or down-regulation was detected for integrins (*Itga6*, *Itga9*, *Itgb1*, *Itgb4*) and laminin (*Lama5*). Based on these results, we expect that the host response to MAP infection at 3 and 6 weeks p.i. could be characterized as an initial innate immune response involving macrophage activation and bactericidal activity against MAP. However, expression of *TNF*, which is probably important for macrophage activation and bactericidal activity together with *IFN-γ*, was not differentially expressed at 3 or 6 weeks p.i. [52,53]. Production of *TNF* has been reported to vary following infection with pathogenic or nonpathogenic mycobacterial species [54,55]. The virulent mycobacterium cell wall component, lipomannan, blocks TNF biosynthesis in macrophages and allows the bacterium to disrupt host immunity [55]. Therefore, low expression of *TNF* was considered to allow MAP to increase its virulence in the host. Additionally, certain genes related to IFN-dependent signaling (such as *Irf7*, *Cxcl9*, *Irgm*, and *Tlr8*) were up-regulated, whereas *Cd69* and other *IRF* genes were down-regulated (Fig 4). Although these up-regulated genes are involved in the innate immune response against intracellular pathogens, down-regulation of *Irf* and *Cd69* genes is considered to induce inactivation of various IFN genes, including *IFN α/β* and function downstream of *IFN α/β* [56]. *Tlr8*, which was known to play a role in the suppression of regulatory T cell activity [57], showed a close linkage with P38 mitogen-activated protein kinase (P38 MAPK), interferon regulatory factors, and *IFN type 1* in this network (Fig 4). Down-regulation of P38 MAPK was thought to induce the inactivity of anti-inflammatory cytokines.

*IFN-γ*, which is known to be essential for mammalian host defense against intracellular pathogens, was a central factor in the common network at 3 and 6 weeks p.i. (Fig 5). Interferon-inducible protein-encoding genes (*Gbp*, *Irgm*) together with *IFN-γ* were up-regulated in this network and may also play a role in the innate immune response by regulating autophagy against intracellular pathogens [45,58]. In particular, IRGM proteins help trigger autophagy in cells infected with mycobacteria, and several polymorphisms in or near *IRGM* have been associated with an increased risk of developing Crohn’s disease [58–60]. In accordance with the down-regulation of *Hla-doa*, *Hla-dob*, and *Hla-dmb*, MHC class II gene expression was down-regulated in MAP-infected mice at 3 and 6 weeks p.i. In addition, macrophage receptor with collagenous structure (*Marco*, 2.2- and 1.9-fold up-regulation at 3 and 6 weeks p.i., respectively) and *Cd14* (2.0- and 5.0-fold up-regulation at 3 and 6 weeks p.i., respectively) were up-regulated, and these genes encode novel products required for TLR signaling, and play a role in the secretion of pro-inflammatory cytokines in response to cell wall glycolipids through cooperation between *Marco* and *Tlr2/Cd14* [61].

On the other hand, mononuclear phagocytes serve as the intracellular niche for mycobacteria survival, replication and evasion of host defense [62,63]. Phagocytes use scavenger receptor class F (*Scarf1*) to identify and engulf apoptotic cells using the complement component *C1q* [64]. In addition, complement opsonization enhances the uptake of bacilli by bovine mononuclear phagocytes [65]. We observed a direct relationship between the complement components *C1q* and *Scarf1* in the network, which suggests that MAP may be taken up by macrophages via *Scarf1* using the complement component *C1q*. Nonopsonic internalization of mycobacteria in macrophages can be mediated by different kinds of receptors that specifically recognize ligands expressed on the surface of bacilli such as glycolipid and phospholipid [66]. Several receptors have been established as putative route of entry for mycobacteria into macrophages including glycophosphoinositol-anchored receptors such as CD14, scavenger receptor, complement receptor 3, and mannose receptor [67–69]. In the present study, mannose receptor (*Mrc1*, 15.4- and 4.8-fold up-regulation at 6 and 12 weeks p.i., respectively) showed significant up-regulation, but scavenger receptor class A (*ScaraA*, 2.6-, 3.3-, and 2.1-fold down-regulation at 3, 6 and 12 weeks p.i., respectively), known as an important mediator of mycobacteria-macrophage interactions [68], was down-regulated.

Among characteristics of M2 macrophage phenotype, down-regulation of *MHC class II* (*Hla-dmb*, 3.2- and 2.3-fold down-regulation; *Hla-doa*, 2.9- and 2.3-fold down-regulation; *Hla-dob*, 5.5- and 4.0-fold down-regulation at 3 and 6 weeks p.i., respectively, *p* < 0.001), *Ccr7* (2.3- and 1.9-fold down-regulation at 3 and 6 weeks p.i., respectively, *p* < 0.001), and *Irf5* (1.7- and 1.6-fold down-regulation at 3 and 6 weeks p.i., respectively, *p* < 0.001), and up-regulation of *Mrc1* (1.7-and 15.4-fold up-regulation at 3 and 6 weeks p.i., respectively, *p* < 0.001), and *MARCO* (2.2- and 1.9-fold up-regulation at 3 and 6 weeks p.i., respectively, *p* < 0.001) were observed in MAP-infected mice at 3 or 6 weeks p.i. [70,71]. In addition, canonical pathways related to the T cell immune response, such as ICOS-ICOSL signaling in T helper cells, calcium-induced T lymphocyte apoptosis, and CD28 signaling in T helper cells, were suggested to be strongly down-regulated at 3 and 6 weeks p.i. Down-regulation of CD28 signaling in T helper cells indicated low T cell activity, because IL–2 production through CD28 signaling affects various immune and non-immune processes, including cell cycle progression, T-cell survival, T-helper cell differentiation, and immunoglobulin isotype switching [72]. In particular, among the downstream genes of canonical pathways related to the T cell immune response, which were down-regulated following the MAP infection, NFAT (nuclear factor of activated T-Cells) and NF-κB (nuclear factor-kappaB) are known to play a key role in the transcription of the *IL–2* gene and other anti-apoptotic genes involved in these pathways [73].

Consequently, the host responses to MAP infection at 3 and 6 weeks p.i. were characterized by an initial innate immune response involving macrophage activation and bactericidal activity against MAP invasion into macrophage via several receptors, although they were also expected to show the M2 macrophage phenotype and down-regulation of signaling related to the T cell immune response. Additionally, although MAP was continuously detected in the spleen and liver by MAP-specific *IS900* PCR during the experimental period from 3 to 16 weeks p.i. (data not shown), reduction of bacilli number in the MAP-infected mice occurred based on reduction of the lesion score, the acid-fast bacilli score, and the number of differentially expressed genes throughout the experimental period. We investigated a mouse model for the MAP infection with immunopathological and transcriptional analysis. These results may provide information for research into the pathogenesis of MAP infection in a murine model.

## Materials and Methods

### Ethics statement

All animal procedures were carried out according to the recommendations and guidelines of Institutional Animal Care and Use Committee (IACUC) of Animal and Plant Quarantine Agency, Korea and the protocol approved by 'Seoul National University Institutional Animal Care and Use Committee (SNUIACUC) (permit no. SNU-120919-6).

### Bacterial strain and animal infection

*Mycobacterium avium* subsp. *paratuberculosis* ATCC 19698 was grown at 37°C on 7H10 agar (per liter) (19 g, BD Biosciences, Sparks, MD, USA) containing a supplement (oleic acid 0.6 g, albumin 50 g, dextrose 20 g, and catalase 0.03 g [OADC enrichment]; Difco Laboratories, Detroit, MI, USA); mycobactin J (2mg, Allied Monitor, Fayette, MO, USA); casitone (1g, BD Biosciences); glycerol (5ml); Bactec Meit PANTA antibiotic mixture (Polymyxin B 6,000 units, Trimethoprim 600 μg, Amphotericin B 600 μg, Azlocillin 600 μg, Nalidixic acid 2,400 μg; BD Biosciences); and egg yolk (250ml). For animal infection, MAP ATCC 19698 was suspended in phosphate-buffered saline (PBS) and used at a dilution of 1 × 10^9^ cells/mL.

Five-week-old C57BL/6 female mice (OrientBio Co. Ltd., Kyunggi-do, Republic of Korea) were used, and were provided with standard mouse food and water *ad libitum*. All mice were cared for according to the policy and regulations for the care and use of laboratory animals of the Laboratory Animal Center and Seoul National University, Korea. Mice were intraperitoneally inoculated with 300 μL of PBS (control group) or a suspension of MAP ATCC 19698 (3 × 10^7^ cells/mouse, MAP-challenged group). Five mice from each group were weighed and killed at 3, 6, 9, 12, and 16 weeks post-infection (p.i.) [19,20,74]. The carcasses and excised livers and spleens were weighed and collected for histopathological examination. Among them, two mice were randomly chosen and their spleens were used for microarray analysis.

### Histopathological findings and acid-fast stain

After necropsy, three to four sections from the liver and the spleen of each mouse at each time point were collected and fixed in 10% phosphate-buffered formalin. For histopathological examination, formalin-fixed tissues were processed using routine methods, embedded in paraffin, and stained with hematoxlyin and eosin (HE). Replicate sections from the liver and spleen were also stained with Ziehl-Neelsen’s acid-fast stain. Severity of the lesions and the relative number of acid-fast bacilli were graded as 0 if absent, and from +1 to +4 if present based on the extent of granulomatous inflammation and presence of acid-fast bacilli with five fields per mouse, respectively, as previously described [15]. The mean lesion score of either the liver or spleen at 3, 6, 9, 12, and 16 weeks p.i. was compared statistically using Student’s *t*-test. The data were expressed as the mean ± standard error (SE), and statistical significance was analyzed using a nonparametric test (Mann-Whitney U test).

### RNA preparation, labeling and purification

Total RNA was isolated from the spleens of experimental mice using RNeasy Mini Kit (Qiagen, Valencia, CA, USA) according to the manufacturers’ protocol. After processing with DNase digestion, and clean-up procedures, RNA samples were quantified, aliquoted and stored at -80°C until use. For quality control, RNA purity and integrity were evaluated by denaturing gel electrophoresis, OD 260/280 ratio, and analyzed on an Agilent 2100 Bioanalyzer (Agilent Technologies, Palo Alto, CA, USA). Total RNA was amplified and purified using an Ambion Illumina RNA amplification kit (Ambion, Austin, TX, USA) to yield biotinylated cRNA according to the manufacturer’s instructions. Briefly, 550 ng of total RNA was reverse-transcribed to cDNA using a T7 oligo(dT) primer. Second-strand cDNA was synthesized, *in vitro* transcribed, and labeled with biotin-NTP. After purification, the cRNA was quantified using an ND–1000 Spectrophotometer (NanoDrop, Waltham, MA, USA) for confirmation of RNA purity and integrity with a mean A_260_/A_280_ ratio of 2.03±0.02 and mean RNA integrity number of 7.05±0.28, respectively.

### Hybridization and data export

Labeled cRNA samples (750 ng) were hybridized to each Mouse WG6 expression v.2 bead array for 16–18 h at 58°C, according to the manufacturer's instructions (Illumina, Inc., San Diego, CA, USA). Detection of array signal was carried out using Amersham fluorolink streptavidin-Cy3 (GE Healthcare Bio-Sciences, Little Chalfont, UK) following the bead array manual. Arrays were scanned with an Illumina bead array reader confocal scanner according to the manufacturer's instructions

### Raw data preparation and statistical analysis

The quality of hybridization and overall chip performance were monitored by visual inspection of both internal quality control checks and the raw scanned data. Raw data were extracted using the software provided by the manufacturer (Illumina GenomeStudio v2011.1 (Gene Expression Module v1.9.0)).

Array probes were transformed by logarithm and normalized by the quantile method. After normalization, probes corrected batch effects using ComBat (http://www.bu.edu/jlab/wp-assets/ComBat/Abstract.html). Statistical significance of the expression data was determined using LPE test and fold change with the null hypothesis that no difference exists among groups. False discovery rate (FDR) was controlled by adjusting *p*-value using the Benjamini-Hochberg algorithm. A ≥2-fold change in expression (for both up- and down-regulation) and an adjusted *p* value of less than 0.05 were used as cutoffs to determine differential expression. For a DEG set, hierarchical cluster analysis was performed using complete linkage and Euclidean distance as a measure of similarity.

Gene-Enrichment and Functional Annotation analysis of the significant probe list was performed using DAVID (http://david.abcc.ncifcrf.gov/home.jsp).

All data analysis and visualization of differentially expressed genes were conducted using R 3.0.2 (www.r-project.org).

### Biological system analysis

Data were analyzed using Ingenuity Pathway Analysis (IPA; Ingenuity Systems Inc., Redwood, CA, USA) for canonical pathways and functional processes. Differentially expressed genes with adjusted *p* values less than 0.05 showing a 2-fold or greater change (up-regulation or down-regulation) were uploaded into the IPA program. Each gene was mapped to its corresponding gene object in Ingenuity’s Knowledge Base. Biological function analysis was performed using IPA to compare the diseases and disorders, molecular and cellular functions, and physiological system development and function of differentially expressed genes in MAP-challenged mice. A right-tailed Fisher’s exact test was adopted to calculate the *p*-value for each biological function. Canonical pathways from the IPA library of canonical pathways were investigated to identify major biological pathways associated with MAP infection in mice. The significance of association between the data set and canonical pathway was determined based on two parameters: (1) The ratio of the number of genes from the data set that map to the pathway to the total number of genes that map to the canonical pathway and (2) *p*-value calculated using Fisher's exact test determining the probability that the association between the genes in the data set and the canonical pathway results from chance alone.

### qPCR validation

To validate the microarray results, we analyzed six selected genes showing differential expression using quantitative real-time PCR (qRT-PCR, Table 2). qRT-PCR reactions were performed with 1 μL of cDNA using a Rotor-Gene SYBR Green PCR kit (Qiagen) and Rotor-Gene Q real-time PCR cycler (Qiagen). Amplification was performed for 35 cycles at 95°C for 15 sec, followed by 45 sec at 60°C with fluorescence detected during the extension phase. Expression level was determined by the 2^-ΔΔCt^ method using glyceraldehyde-3-phosphate dehydrogenase (GAPDH) as a reference gene. The relative expression level was compared to that in control mice to determine the fold change in expression for each gene.

### Statistical analysis

The data were expressed as mean ± SE and statistical significance was analyzed by Student’s *t*-test. Histopathological scores, a nonparametric test (Mann-Whitney U test) was performed using Statistical Package for Social Sciences software (SPSS, version 20, SPSS Inc., Chicago, IL, USA). Pearson’s correlation coefficient was calculated for logarithmically transformed data obtained using qPCR and the microarray. Differences were considered significant when *p* < 0.05.

## Supporting Information

S1 Table The 10 most up- or down-regulated genes in the MAP-infected mice. (DOCX)
