# Supplementary material for: Host Transcriptional Profiles and Immunopathologic Response following Mycobacterium avium subsp. paratuberculosis Infection in Mice
Source: PLoS One. 2015 Oct 6;10(10):e0138770. doi: 10.1371/journal.pone.0138770 (PMC4595071; doi:10.1371/journal.pone.0138770)
Supplement: S1 Table — (DOCX) [file pone.0138770.s001.docx]

**S1 Table. The 10 most up- or down-regulated genes in the MAP-infected mice**

|  | **Gene symbol** | **RefSeq** | **Description** | **Fold-change** |
| --- | --- | --- | --- | --- |
| At 3 weeks p.i. | | | | |
| up | *Saa3* | NM_011315.3 | serum amyloid A 3 | 70.54 |
|  | *A330102K04Rik* | XR_001572.1 | PREDICTED: Mus musculus RIKEN cDNA A330102K04 gene | 35.01 |
|  | *Gbp1* | NM_010259.2 | guanylate binding protein 1 | 19.01 |
|  | *Kel* | NM_032540.2 | Kell blood group | 14.97 |
|  | *Ank1* | NM_031158.1 | ankyrin 1, erythroid | 14.88 |
|  | *Gpnmb* | NM_053110.3 | glycoprotein (transmembrane) | 14.22 |
|  | *Abcg4* | NM_138955.3 | ATP-binding cassette, sub-family G (WHITE), member 4 | 13.97 |
|  | *LOC100046690* | XM_001476775.1 | PREDICTED: Mus musculus hypothetical protein LOC100046690 | 13.88 |
|  | *Hemgn* | NM_053149.2 | hemogen | 13.86 |
|  | *Gypa* | NM_010369.3 | glycophorin A | 13.57 |
| down | *Cfd* | NM_013459.1 | complement factor D (adipsin) | -12.35 |
|  | *Ly6d* | NM_010742.1 | lymphocyte antigen 6 complex, locus D | -9.45 |
|  | *Gm459* | NM_010742.1 | PREDICTED: Mus musculus gene model 459, (NCBI) | -8.15 |
|  | *Vpreb3* | XM_992644.1 | pre-B lymphocyte gene 3 | -7.33 |
|  | *LOC384415* | NM_009514.4 |  | -7.24 |
|  | *LOC100038894* | XM_357633.1 | PREDICTED: Mus musculus hypothetical protein LOC100038894 | -6.79 |
|  | *LOC100047815* | XM_001471776.1 | PREDICTED: Mus musculus similar to CD79A antigen (immunoglobulin-associated alpha) | -6.77 |
|  | *Blk* | XM_001478920.1 | B lymphoid kinase | -6.70 |
|  | *Sbk* | NM_007549.2 |  | -6.49 |
|  | *Cd79b* | NM_145587.1 | CD79B antigen | -6.48 |
| At 6 weeks p.i. | | | | |
| up | *A330102K04Rik* | XR_001572.1 | PREDICTED: Mus musculus RIKEN cDNA A330102K04 gene | 67.74 |
|  | *Gypa* | NM_010369.3 | glycophorin A | 65.98 |
|  | *S100a8* | NM_013650.2 | S100 calcium binding protein A8 (calgranulin A) | 64.76 |
|  | *Ltf* | NM_008522.3 | lactotransferrin | 64.27 |
|  | *Trim10* | NM_011280.1 | tripartite motif-containing 10 | 63.59 |
|  | *Lcn2* | NM_008491.1 | lipocalin 2 | 59.55 |
|  | *Car1* | NM_009799.4 | carbonic anhydrase 1 (Car1), transcript variant 1 | 47.42 |
|  | *S100a9* | NM_009114.1 | S100 calcium binding protein A9 (calgranulin B) | 46.77 |
|  | *Hbb-b1* | AK005442 | hemoglobin, beta adult major | 45.49 |
|  | *Ctse* | NM_007799.2 | cathepsin E | 41.42 |
| down | *Psmb1* | NM_011185.3 | proteasome (prosome, macropain) subunit, beta type 1 | -8.84 |
|  | *Fcer2a* | NM_013517.1 | Fc receptor, IgE, low affinity II, alpha polypeptide | -6.73 |
|  | *Ccl21c* | NM_023052.1 | chemokine (C-C motif) ligand 21c (leucine) | -5.97 |
|  | *Igfbp5* | NM_010518.2 | insulin-like growth factor binding protein 5 | -5.71 |
|  | *Il7r* | NM_008372.3 |  | -5.40 |
|  | *LOC100041504* | XM_001473258.1 | PREDICTED: Mus musculus similar to beta chemokine Exodus-2 | -5.37 |
|  | *Vpreb3* | NM_009514.4 | pre-B lymphocyte gene 3 | -5.16 |
|  | *Cd37* | NM_007645.2 | CD37 antigen | -5.16 |
|  | *Ebi2* | NM_183031.1 | Epstein-Barr virus-induced gene 2 | -5.02 |
|  | *Chst3* | NM_016803.2 | carbohydrate (chondroitin 6/keratan) sulfotransferase 3 | -4.95 |
| At 12 weeks p.i. | | | | |
| up | *Hbb-b1* | AK005442 | hemoglobin, beta adult major chain | 5.27 |
|  | *Fam132a* | NM_026125.2 | family with sequence similarity 132, member A | 4.802 |
|  | *Rhag* | NM_011269.1 | Rhesus blood group-associated A glycoprotein | 4.42 |
|  | *Tal1* | NM_011527.1 | T-cell acute lymphocytic leukemia 1 | 4.24 |
|  | *EG240327* | NM_001033767.2 | predicted gene, EG240327 | 3.95 |
|  | *Gclm* | NM_008129.3 | glutamate-cysteine ligase , modifier subunit | 3.73 |
|  | *Prdx2* | NM_011563.2 | peroxiredoxin 2 | 3.10 |
|  | *Ear4* | NM_001017422.1 | eosinophil-associated, ribonuclease A family, member 4 | 3.03 |
|  | *Gdf3* | NM_008108.4 | growth differentiation factor 3 | 2.64 |
|  | *Igh-VJ558* | XM_354700 | PREDICTED: Mus musculus immunoglobulin heavy chain (J558 family) | 2.61 |
| down | *LOC383196* | XM_001474081.1 | PREDICTED: Mus musculus hypothetical LOC383196 | -4.15 |
|  | *Igfbp5* | NM_010518.2 | insulin-like growth factor binding protein 5 | -2.89 |
|  | *2300002D11Rik* | NM_001081156.1 | RIKEN cDNA 2300002D11 gene | -2.19 |
|  | *Fcer2a* | NM_013517.1 | Fc receptor, IgE, low affinity II, alpha polypeptide | -2.14 |
|  | *Scara3* | NM_172604.3 | scavenger receptor class A, member 3 | -2.09 |
